# Supplementary material for: Assessment of Herd, Calf, and Colostrum Management Practices on Austrian Dairy Farms Using a Scoring System
Source: Animals (Basel). 2023 Aug 30;13(17):2758. doi: 10.3390/ani13172758 (PMC10486773; doi:10.3390/ani13172758)

Table S1: Original survey (German and translated to English) on herd-, calf- and colostrum management practices on 2,210 Austrian farms. In total 24 single choice questions were included.

Fragebogen Kälbermanagement – Kolostrummanagement

Daniela Klein-Jöbstl und Nicole Hechenberger

1. In welchem Bundesland befindet sich Ihr Betrieb?

    Burgenland

    Kärnten

    Niederösterreich

    Oberösterreich

    Salzburg

    Steiermark

    Tirol

    Vorarlberg

    Wien

2. Sind Sie Mitglied bei (Mehrfachantwort möglich):

    TGD (Tiergesundheitsdienst)

    LKV

    Sonstige ______________________________

3. Tierkategorie

    Mutterkuh

    Milchvieh

4. Wie führen Sie Ihren Betrieb? (Wirtschaftsweise)

    biologisch

    konventionell

5. Betriebsstruktur

    Nebenerwerb

    Haupterwerb

6. Wie viele Milchkühe/ Mutterkühe befinden sich auf Ihrem Betrieb?

    ____

7. GVE (Großvieheinheit)

    ____

8. Welche Rasse befindet sich vorwiegend in Ihrem Betrieb?

    Fleckvieh

    Braunvieh

Holstein (HF rot/schwarz)

Pinzgauer

Sonstige _________________________________

9. Welche Art der Aufstallung haben Sie bei den laktierenden Kühen?

Laufstall und Auslauf/Weide

Laufstall ohne Auslauf/Weide

Anbindehaltung und Auslauf/Weide

Anbindehaltung ohne Auslauf/Weide

Freilandhaltung/ganzjährig Weide

Sonstiges____________________________

10. Nur für Milchviehbetriebe: Wie hoch ist die durchschnittliche Milchleistung Ihrer Herde (305 Tage Standardlaktation)?

____

11. Haben Sie einen eigenen Abkalbebereich (z.B. Abkalbebox)?

ja

nein

11.a. Wieviel Prozent Ihrer Kühe kalben in diesem Abkalbebereich?

alle (100 %)

fast alle (etwa 90 %)

die meisten (etwa 75 %)

etwa die Hälfte

weniger als die Hälfte (< 50 %)

12. Wie lange bleiben die Kälber nach der Geburt gewöhnlich bei der Mutter?

gar nicht bzw. bis Kalb trocken geleckt wurde (< 20 Minuten)

bis zu 1 Stunde

1 bis 4 Stunden nach der Geburt

länger als 4 Stunden bis zu 1 Tag

> 1 Tag

am Betrieb gibt es eine Ammenkuhhaltung

am Betrieb gibt es eine Mutter-gebunde Aufzucht

13. Wann wird die Kuh nach der Kalbung erstmals gemolken

direkt nach der Kalbung, innerhalb einer Stunde

zwischen 1 und 6 Stunden nach der Kalbung

bei der nächsten Melkzeit (Morgens/Abends, egal, wann Kuh kalbt)

die Kuh wird nicht gemolken, das Kalb bleibt bei der Mutter und saugt

14. Wird das Euter vor der Melkung der Biestmilch gereinigt?

ja

nein

wenn ja wie? ________________________________

15. Wie wird Kolostrum ermolken?

Melkmaschine

per Hand

die Kuh wird nicht gemolken, das Kalb bleibt bei der Mutter

16. Wann wird gewöhnlich das erste Mal Biestmilch verabreicht?

innerhalb 1 Stunde nach der Geburt

später als 1 Stunde, innerhalb von 4 Stunden

später als 4 Stunde, innerhalb von 6 Stunden

bei der nächsten Melkzeit (Morgens/Abends, egal, wann Kuh kalbt)

gar nicht, Kalb trinkt am Euter der Mutter

17. Wird das Kalb mit dem Kolostrum der eigenen Mutter getränkt?

ja, immer

ja, meistens

ja, wenn die Mutter gute Kolostrumqualität hat

das Kalb bleibt bei der Mutter, trinkt selbst

nein, nicht immer

18 a. wenn nein, welche Biestmilch bzw. Kolostrumersatz wird verwendet?

____

18. Die Biestmilchmenge innerhalb der ersten 6 Stunden beträgt gewöhnlich:

< 2 Liter

2-4 Liter

> 4- 6 Liter

> 6 Liter

unbekannt, Kalb trinkt bei Kuh

19. Wird die Biestmilchqualität beurteilt?

ja

nein

19.a. Wie beurteilen Sie die Kolostrumqualität?

Kolostrumspindel/Kolostrometer/Biestmilchtester

Refraktometer (optisch oder digital)

Augenschein (Farbe, Fließeigenschaft)

Trichter

Sonstiges _________________

20. Wenn ein Kalb die Biestmilch nicht freiwillig trinkt, wird…:

    das Kalb unmittelbar mit Biestmilch gedrencht.

    das Kalb innerhalb von 2 bis 6 Stunden gedrencht, wenn es bis dahin nicht selbstständig getrunken hat.

    Am Betrieb wird generell gedrencht, egal ob das Kalb selbstständig trinken würde oder nicht.

    Das Kalb wird auf keinen Fall gedrencht, es wird wiederholt Biestmilch angeboten.

    Sonstiges: ______________________________

21. Wie wird dem Kalb das Kolostrum normalerweise verabreicht?

    Eimer

    Trinkflasche

    Drencher

    Kalb bleibt bei der Mutter, trinkt am Euter

22. Haben Sie Biestmilch für Notfälle eingefroren?

    ja

    nein

Survey on colostrum management

Daniela Klein-Jöbstl and Nicole Hechenberger

1. In which province is the farm located?
    a. Burgenland
    b. Carinthia
    c. Lower Austria
    d. Upper Austria
    e. Salzburg
    f. Styria
    g. Tyrol
    h. Vorarlberg
    i. Vienna
2. Are you a member of:
    a. Animal health service
    b. Milk performance recording organisation
    c. Other _______________
3. Animal category:
    a. Cow/calf operation

    b. Dairy cattle
4. What is the production type?
    a. Organic farming
    b. Conventional farming
5. How is the farm operated?
    a. Full-time
    b. Part-time
6. How many dairy cows are kept on the farm? (nCow)
    a. _________________
7. How many livestock units are on the farm?
    a. _________________
8. What breed is primarily kept on your farm?
    a. Simmental
    b. Holstein-Fresian
    c. Original Brown/Brown Swiss
    d. Pinzgauer
    e. Other __________________
9. What is the housing type on your farm for lactating/dry cows?
    a. Freestall barn with outdoor loafing area/pasture
    b. Freestall barn without outdoor loafing area/pasture
    c. Tie stalls with outdoor loafing area/pasture
    d. Tie stalls without outdoor loafing area/pasture
    e. Free range/all year round pasture
    f. Other ______________
10. Only for dairy cattle farms: What is the average milk yield in your herd (305 days standard lactation)?
    a. __________________
11. Do you have a separate calving area?
    a. Yes
    b. No

    11a. How many % of your cattle calve in the separate calving area?
        a. all (100 %)
        b. almost all (90 %)
        c. the majority (75 %)
        d. half (50 %)
        e. less than half (< 50 %)
12. How long do you leave the calf with the dam after birth?
    a. Not at all, until the dam licked the calf dry (< 20 min.)
    b. Up to 1 hour
    c. 1 - 4 hours after birth
    d. More than 4 hours, up to 1 day
    e. > 1 day
    f. Nurse cow calf rearing system
    g. Dam bound calf rearing system
13. When is the dam first milked after calving?
    a. Directly after calving, within 1 hour
    b. Between 1 and 6 hours after calving
    c. At the next standard milking time (in the morning/evening, no matter when the cow calves)

    d. The dam is not being milked, the calf stays with the dam

14. Do you clean the udder before milking colostrum?
    a. Yes
    b. No
    c. If yes, how? ______________________
15. How do you harvest colostrum?
    a. Milking machine
    b. By hand
    c. The dam is not being milked, the calf stays with the dam
16. When is the calf usually fed colostrum for the first time?
    a. Within 1 hour after birth
    b. Between 1 – 4 hours after birth
    c. Between 4 – 6 hours after birth
    d. At the ned standard milking time (in the morning/evening, no matter when the cow calves)
    e. Not at all, the calf is left to suckle the dam
17. Is the calf fed with colostrum from his/her own dam?
    a. Yes, always
    b. Yes, mostly
    c. Yes, if the dam has good colostrum quality
    d. The calf stays with the dam and nurses
    e. No, not always

17a. If no, which colostrum or colostrum replacer is being used?

    a. ___________________________

18. The usual quantity of colostrum within the first 6 hours is:
    a. < 2 litre
    b. 2 – 4 litre
    c. > 4 – 6 litre
    d. > 6 litre
    e. Unknown, the calf is left to suckle the dam
19. Do you test colostrum quality?
    a. Yes
    b. No

19a. If yes, how do you test colostrum quality?

    a. Colostrometer
    b. Refractometer (optical or digital)
    c. visually (colour/fluidity)
    d. ColostroCheck Kritzinger
    e. Other _______________

20. If the calf does not drink colostrum willingly, what do you do?
    a. The calf is immediately fed by oesophageal tube
    b. The calf is fed by oesophageal tube within 2 – 6 hours, if it hasn’t drunk colostrum independently by that time

c. On the farm all calves are fed by oesophageal tube in general, no matter if they would drink willingly or not
d. In no case is the calf fed by oesophageal tube, colostrum is offered multiple times
e. Other ____________________

21. How do you usually feed colostrum to the calf?
    a. Bucket
    b. Nipple bottle
    c. Oesophageal tube
    d. The calf stays with the dam and nurses
22. Do you have frozen colostrum storages in case of emergencies?
    a. Yes
    b. No

Table S2: Allocation of points for the scoring system. The questionnaire was split in three sections. Section 1 was on general farm characteristics (question number 1 to 11), section 2 was on herd management practices (questions number 12 to 19) and section 3 was on calf management practices (questions number 20 to 27). Blue colour in 'Score' column means answers were not included in the scoring system.

Nr.	Category	Question	Answer possibilities	Score	Reference
1	Overview	In which province is the farm located?	Burgenland		
			Carinthia		
			Lower Austria		
			Upper Austria		
			Salzburg		
			Styria		
			Tyrol		
			Vorarlberg		
			Vienna		
2	Overview	Are you a member of the Animal Health Service	Yes		
			No		
3	Overview	Are you a memeber of routine milk recording	Yes		Excluded from study
			No		
4	Overview	Animal Category	dairy cattle		
5	Overview	What is the operation type?	organic farming		
			conventional farming		
6	Overview	How is the farm operated?	Full-time		
			Part-time		
7	Overview	How many dairy cows are kept on the farm? (nCow)	≤ 10		
			11 - 20		
			21 - 30		
			31 - 40		
			≥ 40		
8	Overview	How many livestock units are on the farm?	≤ 20		
			21 - 50		
			51 - 100		
			101 - 150		
			≥ 150		
9	Overview	What breed is primarily kept on the farm?	Simmental		
			Holstein-Fresian		
			Original Brown/Brown Swiss		
			Pinzgauer		
			Jersey		
			Tyrol Grey		
			Others (WBB, Tuxer, Angus)		
			Crossbreed		
10	Overview	What is the housing type on the farm for lactating/dry cows?	Freestall barn with outdoor loafing area/pasture		
			Freestall barn without outdoor loafing area/pasture		
			Tie stalls with outdoor loafing area/pasture		
			Tie stalls without outdoor loafing area/pasture		
11	Overview	What is the average milk yield in your herd (305 days standard lactation)	no or implausible answer		
			2000 - 6500		
			6501 - 7500		
			7501 - 8700		
			8701 - 14.000		

Nr.	Category	Question	Answer possibilities	Score	Reference
12	Herd management	How long do you leave the calf with the dam after birth?	not at all, until the dam licked the calf dry (< 20 min.)		
			up to 1 hour		
			1 - 4 hours after birth		
			more than 4 hours, up to 1 day		
			> 1 Day		
			nurse cow calf rearing		
			dam bound calf rearing		
13	Herd management	How do you harvest colostrum?	milking machine	3	S. Stewart et al. 2005. Preventing Bacterial Contamination and Proliferation During the Harvest, Storage, and Feeding of Fresh Bovine Colostrum
			by hand	1	
			the dam is not being milked, the calf stays with the dam	1	
14	Herd management	Do you have a seperate calving area?	Yes	1	Mee JF (2008) Newborn dairy calf management. Vet Clin North am Food Anim Pract 24:1-17
			No	0	
15	Herd management	How many % of your cattle calve in the seperate calving area?	all (100%)	4	Svensson C, Lundborg K, Emanuelson U, Olsson SO (2003) Morbidity in Swedish dairy calves from birth to 90 days of age and individual calf-level risk factors for infectious diseases. Prev Vet Med. 58:179-197
			almost all (90%)	3	
			the most (75%)	2	
			Half (50%)	1	
			less than half <50%	0	
16	Herd management	When is the dam first milked after calving?	directly after calving, within 1 hour	4	A.L. Beam 2009. Prevalence of Failure of passive transfer of immunity in newborn heifer calves and associated management practices on US dairy operations
			between 1 and 6 hours after calving	3	
			at the next milking time (in the morning/evening, no matter when the cow calves)	1	
			the dam is not being milked, the calf stays with the dam	1	
17	Herd management	Do you have colostrum frozen in case of emergencies?	Yes	1	N. M. Holloway et al. 2001. Serum Immunglobulin G concentrations in calves fed fresh and frozen colostrum
			No	0	
18	Herd management	Do you clean the udder before milking colostrum?	Yes	1	S. Stewart et al. 2005. Preventing Bacterial Contamination and Proliferation During the Harvest, Storage, and Feeding of Fresh Bovine Colostrum
			No	0	
19	Herd management	If the udder is cleaned, how?	wood wool		
			Udder cloth wet		
			Udder cloth dry		
			automaticly (robotic systems)		

Nr.	Category	Question	Answer possibilities	Score	Reference
20	Calf mangagement	Is the calf fed with colostrum from his/her own dam	yes, always	2	
			mostly	1	Priestley et al 2013. Effect of feeding maternal colostrum or plasma-derived
			yes, if the dam has good colostrum quality	3	or colostrum derived colostrum replacer on passive transfer of immunity,
			the calf stays with the dam and nurses	1	health, and performance of preweaning heifer calves
			no, not always	2	
21	Calf mangagement	If not always, what do you use?	frozen colostrum	3	Priestley et al 2013. Effect of feeding maternal colostrum or plasma-derived
			colostrum replacer	1	or colostrum derived colostrum replacer on passive transfer of immunity, health, and performance of preweaning heifer calves
22	Calf mangagement	How do you usually feed the calf colostrum?	bucket	3	
			nipple bottle	3	S.M. Godden 2009. Interaction between feeding method and volume
			esophageal feeder	1	colostrum fed
			the calf stays with the dam and nurses	1	
23	Calf mangagement	Do you test the colostrum quality?	Yes	1	Kruse V et al. 1970. Yield of colostrum and immunglobulin in cattle at first
			No	0	milking after parturition
24	Calf mangagement	If yes, how?	Colostrometer	1	Inadequate method for testing is visual inspection. Other testing methods
			Refractometer (optical or digital)	1	depend on the correct usage. There were scored the same; M-C Bartens et
			Visually	0	al. 2016. Assessment of different methods to estimate bovine colostrum
			ColostroCheck Kritzinger	1	quality on farm
25	Calf mangagement	When is the calf usually fed colostrum for the first time?	within 1 hour after birth	4	
			1 - 4 hours after birth	3	A.L. Beam 2009. Prevalence of Failure of passive transfer of immunity in
			4 - 6 hours after birth	2	newborn heifer calves and associated management practices on US dairy
			at the next milking time (in the morning/evening, no matter when the cow calves)	1	operations
			not at all, the calf is left to suckle the dam	1	
26	Calf mangagement	The usual quantity of colostrum within the first 6 hours is:	< 2 Litre	2	
			2 - 4 Litre	3	D.E.Morin 1997. Effects of quality, Quantity and Timing of Colostrum
			> 4 - 6 Litre	3	Feeding
			> 6 Litre	3	
			unknown, the calf is left to suckle the dam	1	
27	Calf mangagement	If a calf does not drink colostrum willingly, what do you do?	the calf is immediately fed by esophageal feeder		
			the calf is fed with an esophageal feeder within 2 - 6 hours, if it hasn't drunk colostrum independetly by that time		
			on the farm all calves are fed in general by esophageal feeder		
			colostrum is offered multiple times, in no case the calf is fed by esophageal feeder		
			colostrum is offered multiple times, later the calf will be fed by esophageal feeder		
			Others (Call the vet, supplements)		

Table S3: Overview of the answers given in survey section one on general farm characteristics of the 2,210 included dairy farms for each federal state. BGL = Burgenland, CAR = Carinthia, LOAT = Lower Austria, UPAT = Upper Austria, SBG = Salzburg, STY = Styria, T = Tyrol, VBG = Vorarlberg, N.A. = no answer, OLA = outdoor loafing area. *The breed category “brown swiss” also included the “original brown swiss” (Original Braunvieh).

Question	Answer category	N answers per federal state (% within federal state)								
		**BGL**	**CAR**	**LOAT**	**UPAT**	**SBG**	**STY**	**T**	**VBG**	**Total**
**Farm size**	≤ 10	1 (12.5)	18 (11.9)	19 (4.7)	16 (3.6)	74 (25.2)	33 (9.7)	190 (43.6)	32 (26.7)	383 (17.4)
	11 -20	1 (12.5)	62 (41.1)	100 (24.8)	83 (18.5)	107 (36.4)	100 (29.3)	140 (32.1)	39 (32.5)	632 (28.7)
	21 – 30	0 (0.0)	31 (20.5)	115 (28.5)	119 (26.5)	46 (15.6)	101 (29.6)	62 (14.2)	33 (27.5)	507 (23.0)
	31 – 40	0 (0.0)	25 (16.6)	68 (16.8)	90 (20.0)	33 (11.2)	48 (14.1)	19 (4.4)	7 (5.8)	290 (13.2)
	≥ 41	6 (75.0)	15 (9.9)	102 (25.2)	141 (31.4)	34 (11.6)	59 (17.3)	25 (5.7)	9 (7.5)	391 (17.7)
	**Total**	**8 (100)**	**151 (100)**	**404 (100)**	**449 (100.0)**	**294 (100.0)**	**341 (100.0)**	**436 (100.0)**	**120 (100.0)**	**2,203 (100.0)**
**Breed**	Simmental	6 (75.0)	108 (72.0)	370 (91.6)	408 (91.6)	218 (74.1)	260 (76.5)	244 (55.8)	22 (18.3)	1,636 (74.3)
	Holstein Fresian	2 (25.0)	28 (18.7)	17 (4.2)	17 (3.8)	22 (7.5)	30 (8.8)	26 (5.9)	14 (11.7)	156 (7.1)
	Brown Swiss/Original Brown	0 (0.0)	6 (4.0)	16 (4.0)	20 (4.4)	9 (3.1)	46 (13.5)	109 (24.9)	81 (67.5)	287 (13.0)
	Pinzgauer	0 (0.0)	2 (1.3)	1 (0.2)	1 (0.2)	42 (14.3)	2 (0.6)	17 (3.9)	0 (0.0)	65 (3.0)
	Tyrolean Grey	0 (0.0)	0 (0.0)	0 (0.0)	3 (0.7)	1 (0.3)	0 (0.0)	33 (7.6)	0 (0.0)	37 (1.7)
	Others and Crossbreeds	0 (0.0)	6 (4.1)	0 (0.0)	1 (0.2)	2 (0.6)	2 (0.6)	8 (1.8)	3 (2.5)	22 (1.0)
	**Total**	**8 (100.0)**	**150 (100.0)**	**404 (100.0)**	**450 (100.0)**	**294 (100.0)**	**340 (100.0)**	**437 (100.0)**	**120 (100.0)**	**2,203 (100.0)**
**Average milk yield per cow per year in litre**	N.A.	0 (0.0)	9 (6.0)	14 (3.5)	17 (3.8)	16 (5.4)	22 (6.5)	27 (6.2)	9 (7.5)	114 (5.2)
	2,000–6,500	0 (0.0)	34 (22.5)	59 (14.6)	62 (13.7)	106 (35.8)	64 (18.8)	147 (33.5)	22 (18.3)	494 (22.4)
	6,501–7.500	2 (25.0)	37 (24.5)	94 (23.3)	99 (22.0)	85 (28.7)	69 (20.2)	114 (26.0)	31 (25.8)	531 (24.0)
	7,501–8,700	1 (12.5)	34 (22.5)	95 (23.5)	133 (29.5)	52 (17.6)	76 (22.3)	95 (21.6)	37 (30.8)	523 (23.7)
	8,701–14,000	5 (62.5)	37 (24.5)	142 (35.1)	140 (31.0)	37 (12.5)	110 (32.3)	56 (12.8)	21 (17.5)	548 (24.8)
	**Total**	**8 (100.0)**	**151 (100.0)**	**404 (100.0)**	**451 (100.0)**	**296 (100.0)**	**341 (100.0)**	**439 (100.0)**	**120 (100.0)**	**2,210 (100.0)**
**Housing type for lactating and dry cows**	Freestall barn with OLA/pasture	4 (50.0)	75 (49.7)	161 (39.9)	181 (40.2)	144 (49.1)	161 (47.2)	112 (25.8)	55 (45.8)	893 (40.6)
	Freestall barn without OLA/pasture	3 (37.5)	19 (12.6)	178 (44.1)	192 (42.7)	13 (4.4)	78 (22.9)	15 (3.5)	5 (4.2)	503 (22.9)
	Tie stalls with OLA/pasture	1 (12.5)	53 (35.1)	50 (12.4)	49 (10.9)	133 (45.4)	102 (29.9)	296 (68.2)	59 (49.2)	743 (33.8)
	Tie stalls without OLA/pasture	0 (0)	4 (2.6)	15 (3.7)	28 (6.2)	3 (1.0)	0 (0)	11 (2.5)	1 (0.8)	62 (2.8)
	**Total**	**8 (100.0)**	**151 (100.0)**	**404 (100.0)**	**450 (100.0)**	**293 (100.0)**	**341 (100.0)**	**334 (100.0)**	**120 (100.0)**	**2,201 (100.0)**

Table S4: Overview of answers from the second survey section on herd-management practices for each federal state. Part 1 includes the calving area, colostrum harvest and storage procedures in each federal state. BGL = Burgenland, CAR = Carinthia, LOAT = Lower Austria, UPAT = Upper Austria, SBG = Salzburg, STY = Styria, T = Tyrol, VBG = Vorarlberg

Question	Answer category	N answers per federal state (% within federal state)								
		**BGL**	**CAR**	**LOAT**	**UPAT**	**SBG**	**STY**	**T**	**VBG**	**Total**
**Availability of a separate calving area**	Yes	7 (87.5)	99 (65.6)	285 (70.7)	326 (72.6)	147 (49.8)	223 (65.4)	135 (30.9)	48 (40.0)	1,270 (57.6)
	No	1 (12.5)	52 (34.4)	118 (29.3)	123 (27.4)	148 (50.2)	118 (34.6)	302 (69.1)	72 (60.0)	934 (42.4)
	**Total**	**8 (100.0)**	**151 (100.0)**	**403 (100.0)**	**449 (100.0)**	**295 (100.0)**	**341 (100.0)**	**437 (100.0)**	**120 (100.0)**	**2,204 (100.0)**
**Cows actually calving in the separate calving are in %**	All (100%)	3 (42.9)	24 (24.2)	79 (27.7)	103 (31.6)	39 (26.4)	41 (18.4)	37 (27.2)	10 (20.8)	336 (26.4)
	Almost all (90%)	3 (42.9)	45 (45.5)	113 (39.6)	147 (45.1)	59 (39.9)	113 (50.7)	53 (39.0)	19 (39.6)	552 (43.4)
	The most (75%)	1 (14.3)	15 (15.2)	41 (14.4)	37 (11.3)	24 (16.2)	43 (19.3)	19 (14.0)	12 (25.0)	192 (15.1)
	Half (50%)	0 (0.0)	9 (9.1)	26 (9.1)	23 (7.1)	10 (6.8)	15 (6.7)	8 (5.9)	5 (10.4)	96 (7.5)
	Less than Half (< 50%)	0 (0.0)	6 (6.1)	26 (9.1)	16 (4.9)	16 (10.8)	11 (4.9)	19 (14.0)	2 (4.2)	96 (7.5)
	**Total**	**7 (100.0)**	**99 (100.0)**	**285 (100.0)**	**326 (100.0)**	**148 (100.0)**	**223 (100.0)**	**136 (100.0)**	**48 (100.0)**	**1,272 (100.0)**
**Colostrum harvesting method**	Milking machine	4 (50.0)	71 (47.0)	244 (60.8)	264 (58.8)	176 (59.9)	213 (62.8)	270 (61.9)	83 (69.2)	1,325 (60.3)
	By hand	2 (25.0)	73 (48.3)	145 (36.2)	176 (39.2)	106 (36.1)	114 (33.6)	153 (35.1)	33 (27.5)	802 (36.5)
	Calf stays with dam	2 (25.0)	7 (4.6)	12 (3.0)	9 (2.0)	12 (4.1)	12 (3.5)	13 (3.0)	4 (3.3)	71 (3.2)
	**Total**	**8 (100.0)**	**151 (100.0)**	**401 (100.0)**	**449 (100.0)**	**294 (100.0)**	**339 (100.0)**	**436 (100.0)**	**120 (100.0)**	**2.198 (100.0)**
**Availability of frozen colostrum stocks**	Yes	7 (87.5)	134 (88.7)	370 (91.8)	398 (89.0)	228 (78.1)	278 (81.8)	318 (72.6)	83 (69.7)	1,816 (82.6)
	No	1 (12.5)	17 (11.3)	33 (8.2)	49 (11.0)	64 (21.9)	62 (18.2)	120 (27.4)	36 (30.3)	382 (17.4)
	**Total**	**8 (100.0)**	**151 (100.0)**	**403 (100.0)**	**447 (100.0)**	**292 (100.0)**	**340 (100.0)**	**438 (100.0)**	**119 (100.0)**	**2,198 (100.0)**

Table S5: Overview of answers from the second survey section on herd-management practices for each federal state. Part 2 includes calf/dam separation, first milking post partum and udder cleaning in each federal state. BGL = Burgenland, CAR = Carinthia, LOAT = Lower Austria, UPAT = Upper Austria, SBG = Salzburg, STY = Styria, T = Tyrol, VBG = Vorarlberg

Question	Answer category	N answers per federal state (% within federal state)								
		**BGL**	**CAR**	**LOAT**	**UPAT**	**SBG**	**STY**	**T**	**VBG**	**Total**
**Time of calf/dam separation**	Not at all (< 20min.)	2 (25.0)	79 (52.7)	235 (58.3)	273 (60.8)	142 (48.3)	214 (62.8)	254 (58.3)	67 (56.3)	1,266 (57.5)
	Up to 1 hour	2 (25.0)	24 (16.0)	73 (18.1)	69 (15.4)	60 (20.4)	48 (14.1)	96 (22.0)	19 (16.0)	391 (17.8)
	1 – 4 hours	2 (25.0)	15 (10.0)	50 (12.4)	53 (11.8)	28 (9.5)	42 (12.3)	43 (9.9)	15 (12.6)	248 (11.3)
	> 4 hours up to 1 Day	1 (12.5)	16 (10.7)	30 (7.4)	35 (7.8)	35 (11.9)	30 (8.8)	30 (6.9)	11 (9.2)	188 (8.5)
	> 1 Day	1 (12.5)	11 (7.3)	7 (1.7)	14 (3.1)	23 (7.8)	5 (1.5)	10 (2.3)	7 (5.9)	78 (3.5)
	Nurse cow calf rearing	0 (0)	1 (0.7)	2 (0.5)	2 (0.4)	1 (0.3)	0 (0)	0 (0)	0 (0)	6 (0.3)
	Dam bound calf rearing	0 (0)	4 (2.7)	6 (1.5)	3 (0.7)	5 (1.7)	2 (0.6)	3 (0.7)	0 (0)	23 (1.1)
	**Total**	**8 (100.0)**	**150 (100.0)**	**403 (100.0)**	**449 (100.0)**	**294 (100.0)**	**341 (100.0)**	**436 (100.0)**	**119 (100.0)**	**2,200 (100.0)**
**Colostrum harvest after partuition**	Within 1 hour	2 (25.0)	83 (55.7)	229 (56.7)	261 (58.0)	161 (54.8)	175 (51.3)	244 (56.0)	61 (51.7)	1,216 (55.3)
	1 – 6 hours	2 (25.0)	51 (34.2)	130 (32.2)	136 (30.2)	99 (33.7)	107 (31.4)	148 (33.9)	48 (40.7)	721 (32.8)
	Next milking time	3 (37.5)	9 (6.0)	39 (9.7)	48 (10.7)	29 (9.9)	54 (15.8)	37 (8.5)	9 (7.6)	228 (10.4)
	Calf stays with dam	1 (12.5)	6 (4.0)	6 (1.5)	5 (1.1)	5 (1.7)	5 (1.5)	7 (1.6)	0 (0.0)	35 (1.6)
	**Total**	**8 (100.0)**	**149 (100.0)**	**404 (100.0)**	**450 (100.0)**	**294 (100.0)**	**341 (100.0)**	**436 (100.0)**	**118 (100.0)**	**2,200 (100.0)**
**Udder cleaning before colostrum milking**	Yes	7 (87.5)	126 (84.0)	351 (86.9)	398 (88.4)	254 (86.4)	313 (91.8)	364 (83.9)	87 (73.1)	1,900 (86.4)
	No	1 (12.5)	24 (16.0)	53 (13.1)	52 (11.6)	40 (13.6)	28 (8.2)	70 (16.1)	32 (26.9)	300 (13.6)
	**Total**	**8 (100.0)**	**150 (100.0)**	**404 (100.0)**	**450 (100.0)**	**294 (100.0)**	**341 (100.0)**	**434 (100.0)**	**119 (100.0)**	**2,200 (100.0)**
**Udder cleaning methods**	Wood wool	0 (0.0)	21 (16.7)	58 (16.6)	105 (26.6)	93 (36.8)	56 (18.0)	43 (11.9)	6 (6.9)	382 (20.2)
	Udder cloth wet	3 (42.9)	50 (39.7)	116 (33.2)	100 25.3)	66 (26.1)	118 (37.9)	124 (34.3)	41 (47.1)	618 (32.7)
	Udder cloth dry	4 (57.1)	52 (41.3)	168 (48.1)	181 (45.8)	90 (35.6)	135 (43.4)	189 (52.4)	39 (44.8)	858 (45.4)
	Automatic (robotic system)	0 (0.0)	3 (2.4)	7 (2.0)	9 (2.3)	4 (1.6)	2 (0.6)	5 (1.4)	1 (1.1)	31 (1.6)
	**Total**	**7 (100.0)**	**126 (100.0)**	**349 (100.0)**	**395 (100.0)**	**253 (100.0)**	**311 (100.0)**	**361 (100.0)**	**87 (100.0)**	**1,889 (100.0)**

Table S6: Overview of answers given in survey section three on calf management practices for each federal state. Part 1 concerning colostrum source and testing methods of colostrum quality. BGL = Burgenland, CAR = Carinthia, LOAT = Lower Austria, UPAT = Upper Austria, SBG = Salzburg, STY = Styria, T = Tyrol, VBG = Vorarlberg

Question	Answer category	N answers per federal state (% within federal state)								
		**BGL**	**CAR**	**LOAT**	**UPAT**	**SBG**	**STY**	**T**	**VBG**	**Total**
**Colostrum from mother**	Yes, always	5 (62.5)	98 (64.9)	244 (60.4)	263 (58.6)	213 (72.7)	227 (66.6)	312 (71.4)	98 (81.7)	1,460 (66.3)
	Mostly	0 (0.0)	3 (2.0)	3 (0.7)	4 (0.9)	3 (1.0)	0 (0.0)	2 (0.5)	0 (0.0)	15 (0.7)
	Yes, if dam has good colostrum quality	1 (12.5)	39 (25.8)	133 (32.9)	163 (36.3)	61 (20.8)	97 (28.4)	105 (24.0)	18 (15.0)	617 (28.0)
	Calf stays with dam	2 (25.0)	10 (6.6)	16 (4.0)	9 (2.0)	15 (5.1)	15 (4.4)	15 (3.4)	4 (3.3)	86 (3.9)
	No, not always	0 (0.0)	1 (0.7)	8 (2.0)	10 (2.2)	1 (0.3)	2 (0.6)	3 (0.7)	0 (0.0)	25 (1.1)
	**Total**	**8 (100.0)**	**151 (100.0)**	**404 (100.0)**	**449 (100.0)**	**293 (100.0)**	**338 (100.0)**	**437 (100.0)**	**120 (100.0)**	**2,203 (100.0)**
**Colostrum source if not from mother**	Frozen colostrum	0 (0.0)	6 (100.0)	16 (100.0)	16 (94.1)	6 (85.7)	4 (100.0)	9 (100.0)	2 (100.0)	59 (96.7)
	Colostrum replacer	0 (0.0)	0 (0.0)	0 (0.0)	1 (5.9)	1 (14.3)	0 (0.0)	0 (0.0)	0 (0.0)	2 (3.3)
	**Total**	**0 (0.0)**	**6 (100.0)**	**16 (100.0)**	**17 (100.0)**	**7 (100.0)**	**4 (100.0)**	**9 (100.0)**	**2 (100.0)**	**61 (100.0)**
**Assessment of colostrum quality**	Yes	2 (25.0)	50 (33.1)	91 (22.5)	139 (30.8)	78 (26.4)	86 (25.2)	112 (25.5)	32 (26.7)	590 (26.7)
	No	6 (75.0)	101 (66.9)	313 (77.5)	312 (69.2)	218 (73.6)	255 (74.8)	327 (74.5)	88 (73.3)	1,620 (73.3)
	**Total**	**8 (100.0)**	**151 (100.0)**	**404 (100.0)**	**451 (100.0)**	**296 (100.0)**	**341 (100.0)**	**439 (100.0)**	**120 (100.0)**	**2,210 (100.0)**
**Colostrum quality assessment method**	Colostrometer	0 (0.0)	0 (0.0)	7 (7.9)	11 (8.1)	11 (14.5)	10 (11.9)	8 (7.2)	4 (12.9)	51 (8.8)
	Refractometer	0 (0.0)	4 (8.2)	18 (20.2)	33 (24.3)	19 (25.0)	20 (23.8)	12 (10.8)	0 (0.0)	106 (18.3)
	Visually	2 (100.0)	45 (91.8)	63 (70.8)	60 (44.1)	40 (52.6)	53 (63.1)	78 (70.3)	27 (87.1)	368 (63.7)
	ColostroCheck	0 (0.0)	0 (0.0)	1 (1.1)	32 (23.5)	6 (7.9)	1 (1.2)	13 (11.7)	0 (0.0)	53 (9.2)
	**Total**	**2 (100.0)**	**49 (100.0)**	**89 (100.0)**	**136 (100.0)**	**76 (100.0)**	**84 (100.0)**	**111 (100.0)**	**31 (100.0)**	**578 (100.0)**

Table S7: Overview of answers given in survey section three on calf management practices for each federal state. Part 2 concerning feeding time, quantity of colostrum and feeding methods. BGL = Burgenland, CAR = Carinthia, LOAT = Lower Austria, UPAT = Upper Austria, SBG = Salzburg, STY = Styria, T = Tyrol, VBG = Vorarlberg, p.n. = post natum

Question	Answer category	N answers per federal state (% within federal state)								
		**BGL**	**CAR**	**LOAT**	**UPAT**	**SBG**	**STY**	**T**	**VBG**	**Total**
**Time from parturition to colostrum feeding**	Within 1 hour p.n.	3 (37.5)	91 (60.7)	240 (60.0)	271 (60.2)	190 (64.8)	178 (52.4)	259 (59.7)	60 (50.0)	1,292 (58.9)
	1 – 4 hours p.n.	3 (37.5)	44 (29.3)	126 (31.5)	134 (29.8)	72 (24.6)	103 (30.3)	133 (30.6)	47 (39.2)	662 (30.2)
	4 – 6 hours p.n.	0 (0.0)	2 (1.3)	4 (1.0)	6 (1.3)	7 (2.4)	13 (3.8)	11 (2.5)	7 (5.8)	50 (2.3)
	Next standard milking time	0 (0.0)	4 (2.7)	12 (3.0)	32 (7.1)	13 (4.4)	33 (9.7)	22 (5.1)	4 (3.3)	120 (5.5)
	Calf suckles the dam	2 (25.0)	9 (6.0)	18 (4.5)	7 (1.6)	11 (3.8)	13 (3.8)	9 (2.1)	2 (1.7)	71 (3.2)
	**Total**	**8 (100.0)**	**150 (100.0)**	**400 (100.0)**	**450 (100.0)**	**293 (100.0)**	**340 (100.0)**	**434 (100.0)**	**120 (100.0)**	**2,195 (100.0)**
**Quantity of colostrum fed within first 6 hours after birth**	< 2 litres	2 (25.0)	34 (22.5)	78 (19.3)	87 (19.3)	27 (9.2)	63 (18.5)	93 (21.3)	28 (23.3)	412 (18.7)
	2 – 4 litres	3 (37.3)	99 (65.6)	292 (72.3)	330 (73.3)	217 (73.8)	243 (71.3)	287 (65.8)	77 (64.2)	1,548 (70.2)
	> 4 – 6 litres	2 (25.0)	10 (6.6)	17 (4.2)	24 (5.3)	38 (12.9)	23 (6.7)	41 (9.4)	12 (10.0)	167 (7.6)
	> 6 litres	0 (0.0)	1 (0.7)	0 (0.0)	1 (0.2)	3 (1.0)	4 (1.2)	5 (1.1)	2 (1.7)	16 (0.7)
	Unknown, calf with dam	1 (12.5)	7 (4.6)	17 (4.2)	8 (1.8)	9 (3.1)	8 (2.3)	10 (2.3)	1 (0.8)	61 (2.8)
	**Total**	**8 (100.0)**	**151 (100.0)**	**404 (100.0)**	**450 (100.0)**	**294 (100.0)**	**341 (100.0)**	**436 (100.0)**	**120 (100.0)**	**2,204 (100.0)**
**Colostrum feeding equipment**	Bucket	3 (37.5)	42 (27.8)	122 (30.4)	140 (31.2)	179 (61.3)	101 (29.8)	239 (54.6)	64 (53.8)	890 (40.5)
	Nipple bottle	4 (50.0)	98 (64.9)	260 (64.8)	301 (67.0)	92 (31.5)	223 (65.8)	173 (39.5)	49 (41.2)	1,200 (54.6)
	Oesophageal tube	0 (0.0)	0 (0.0)	0 (0.0)	0 (0.0)	3 (1.0)	1 (0.3)	8 (1.8)	3 (2.5)	15 (0.7)
	Calf stays with dam	1 (12.5)	11 (7.3)	19 (4.7)	8 (1.8)	18 (6.2)	14 (4.1)	18 (4.1)	3 (2.5)	92 (4.2)
	**Total**	**8 (100.0)**	**151 (100.0)**	**401 (100.0)**	**449 (100.0)**	**282 (100.0)**	**339 (100.0)**	**438 (100.0)**	**119 (100.0)**	**2,197 (100.0)**
**Calves not drinking well, receive colostrum**	Immediately oesophageal tube	1 (14.3)	16 (10.7)	36 (9.0)	73 (16.3)	22 (7.6)	43 (12.6)	38 (8.8)	4 (3.4)	233 (10.6)
	Oesophageal tube within 2–6 hours	2 (28.6)	34 (22.7)	92 (22.9)	118 (26.3)	61 (21.0)	65 (19.1)	69 (15.9)	22 (18.5)	463 (21.1)
	By oesophageal tube in general	0 (0.0)	3 (2.0)	0 (0.0)	0 (0.0)	5 (1.7)	2 (0.6)	10 (2.3)	0 (0.0)	20 (0.9)
	Colostrum offered multiple times, in no case oesophageal tube	3 (42.9)	94 (62.7)	266 (66.2)	254 (56.6)	199 (68.4)	219 (64.4)	308 (71.0)	93 (78.2)	1,436 (65.5)
	Colostrum offered multiple times, later oesophageal tube	1 (14.3)	3 (2.0)	8 (2.0)	2 (0.4)	4 (1.4)	11 (3.2)	8 (1.8)	0 (0.0)	37 (1.7)
	Others (vet, supplements, etc.)	0 (0.0)	0 (0.0)	0 (0.0)	2 (0.4)	0 (0.0)	0 (0.0)	1 (0.2)	0 (0.0)	3 (0.1)
	**Total**	**7 (100.0)**	**150 (100.0)**	**402 (100.0)**	**449 (100.0)**	**291 (100.0)**	**340 (100.0)**	**434 (100.0)**	**119 (100.0)**	**2,192 (100.0)**

Figure S1: Overview on the differences between herd management scores (left figure) and calf management scores (right figure). Each node shows the sample average rank of the federal state. The abbreviations describe the following federal states of Austria: BGL = Burgenland, CAR = Carinthia, LOAT = Lower Austria, UPAT = Upper Austria, SBG = Salzburg, STY = Styria, T = Tyrol, VBG = Vorarlberg. The level of significance was set at p <0.05. All statistically significant values are shown with a red coloured line (p<0.05); all not statistically significant differences are shown in green colour (p≥0.05).

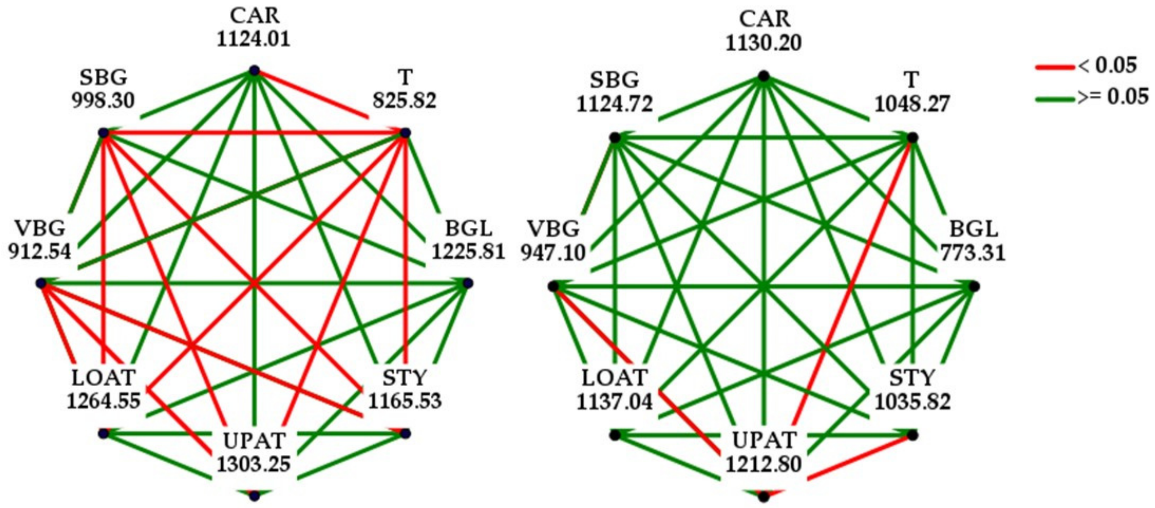

Supplement: Supplementary file 1 [file animals-13-02758-s001.zip › animals-2562572-supplementary.pdf]
